# The overexpression of DNA repair genes in invasive ductal and lobular breast carcinomas: Insights on individual variations and precision medicine

**DOI:** 10.1371/journal.pone.0247837

**Published:** 2021-03-04

**Authors:** Ruwaa I. Mohamed, Salma A. Bargal, Asmaa S. Mekawy, Iman El-Shiekh, Nurcan Tuncbag, Alaa S. Ahmed, Eman Badr, Menattallah Elserafy

**Affiliations:** 1 Center for Informatics Sciences (CIS), Nile University, Giza, Egypt; 2 Center for Genomics, Helmy Institute for Medical Sciences, Zewail City of Science and Technology, Giza, Egypt; 3 University of Science and Technology, Zewail City of Science and Technology, Giza, Egypt; 4 Graduate School of Informatics, Department of Health Informatics, Middle East Technical University, Ankara, Turkey; 5 Faculty of Computers and Artificial Intelligence, Cairo University, Giza, Egypt; University of Ulsan College of Medicine, REPUBLIC OF KOREA

## Abstract

In the era of precision medicine, analyzing the transcriptomic profile of patients is essential to tailor the appropriate therapy. In this study, we explored transcriptional differences between two invasive breast cancer subtypes; infiltrating ductal carcinoma (IDC) and lobular carcinoma (LC) using RNA-Seq data deposited in the TCGA-BRCA project. We revealed 3854 differentially expressed genes between normal ductal tissues and IDC. In addition, IDC to LC comparison resulted in 663 differentially expressed genes. We then focused on DNA repair genes because of their known effects on patients’ response to therapy and resistance. We here report that 36 DNA repair genes are overexpressed in a significant number of both IDC and LC patients’ samples. Despite the upregulation in a significant number of samples, we observed a noticeable variation in the expression levels of the repair genes across patients of the same cancer subtype. The same trend is valid for the expression of miRNAs, where remarkable variations between patients’ samples of the same cancer subtype are also observed. These individual variations could lie behind the differential response of patients to treatment. The future of cancer diagnostics and therapy will inevitably depend on high-throughput genomic and transcriptomic data analysis. However, we propose that performing analysis on individual patients rather than a big set of patients’ samples will be necessary to ensure that the best treatment is determined, and therapy resistance is reduced.

## Introduction

Breast cancer is the most frequent cancer in women and the second most common cancer overall. Incidence rates vary worldwide from 19.3 per 100,000 women in Eastern Africa to 89.7 per 100,000 women in Western Europe [1]. In Egypt, it is the most prevalent cancer among women (35.1%), and the second within the entire population (17.9%) [2]. Breast cancer is a widely heterogeneous disease as it is classified into multiple subtypes varying in clinical and molecular behavior, consequently leading to distinct prognosis and treatment inferences [3]. The most common histological subtypes of breast cancer are the invasive infiltrating ductal carcinoma (IDC) and lobular carcinoma (LC). Their incidence among women account for approximately 80% in case of IDC and 15% in LC [4,5].

IDC and LC differ on the molecular level and clinicopathological features. 90% of LC patients express estrogen receptor (ER) more often than IDC. In addition, 50–70% of LC patients are progesterone receptor positive (PR+), which is higher than IDC. Yet, only 10% of LC are human epidermal growth factor receptor 2 positive (HER2+) [5]. LC also displays a distinct genomic profile when compared to IDC. This includes a higher frequency of CDH1, PIK3CA, HER2, HER3, TBX3 and FOXA1 mutations in addition to more frequent loss of phosphatase and tensin homolog (PTEN) and amplifications of ESR1 [6–8]. A differential gene expression pattern between the two subtypes was also detected. Genes related to cell adhesion and invasion are among the most significant differentially expressed genes between the two subtypes [9]. LC has lower expression of E-cadherin; encoded by CDH1, which is considered the most significant discriminator between the two subtypes. E-cadherin plays a role in cell adhesion, growth and migration and is regarded as a tumor suppressor in breast cancer [10]. This suggests that the two subtypes may follow diverse mechanisms for invasive growth, where it is promoted in LC by the loss of E-cadherin, while in IDC by the overexpression of cathepsins and osteopontin, and down-regulation of thrombospondin [4]. The expression of more genes; ERBB2, p21, SORBS1, VWF, AOC3, MMRN, ITGA7, CD36, and ANXA1, were also found to be discriminative between LC and IDC [5]. On the other hand, well-differentiated IDC and LC express some genes involved in proliferation and cell cycle (cyclin D1, p16 and p27), apoptosis (MIB1 and BCL2), hypoxia response (HIF-1 alpha) and DNA damage response (MDM2) in a similar manner [11–13].

Defects in DNA repair proteins have long been associated with the development and progression of breast cancer [14]. Matta et al. has shown that DNA repair capacity in women with breast cancer reduces by 60% [15]. Around 25–40% of breast cancers have deficiency in homologous recombination (HR) and approximately 25% have Fanconi anemia-BRCA (FA-BRCA) repair pathway deficiency [16]. Moreover, 0.8–1.7% of breast cancers in women show defects in mismatch repair pathway (MMR) [17,18]. Single nucleotide polymorphisms (SNPs) in the XRCC1 and APE1 base excision repair (BER) genes have also been reported to increase the risk of breast cancer on the level of individuals. However, no population specific data was reported [19,20]. Nucleotide excision repair (NER) deficiency has also been associated with early stage breast cancer. It increases breast cancer risk in women exposed to cigarette smoke [21,22]. Furthermore, the ATM mutations account for more than 7% of breast cancer patients. It was additionally estimated that heterozygous carriers of ATM mutations have a two-fold higher risk of breast cancer [23–25].

Despite the rigorous analyses of DNA repair genes in breast cancer, the transcriptomic changes of DNA repair genes in IDC and LC are not well studied. In this study, we focused on transcriptomic analysis of IDC and LC patient tumors to reveal the expression profile of DNA repair genes in these two breast cancer subtypes. Despite showing distinct transcriptomic profiles, to our surprise, the DNA repair genes were similarly expressed in both IDC and LC. Nevertheless, we stress on the importance of taking individual variations into consideration upon analyzing DNA repair genes in cancer patients.

## Methodology

### Data retrieval

Both RNA sequencing (RNA-seq) and microRNA sequencing (miRNA-seq) data were retrieved from The Cancer Genome Atlas—Breast Invasive Carcinoma (TCGA-BRCA) project (accession date: March 05, 2020) in the format of HTSeq-Counts for RNA-seq and BCGSC miRNA Profiling text files for miRNA-seq. The focus of this study is on females with ductal and lobular neoplasms. We further selected the cases with either invasive ductal carcinoma (IDC) or lobular carcinoma (LC) using the clinical datasheet of the TCGA-BRCA project. The data included 947 cases with both RNA-seq and miRNA-seq data, and 17 cases with RNA-seq data only (total of 964 cases). The 964 cases contributed 1,063 RNA-seq samples (89 normal_ductal, 771 IDC, and 203 LC) and 1,044 miRNA-seq samples (82 normal_ductal, 760 IDC, and 202 LC). Note that 7 RNA-seq and 6 miRNA-seq normal lobular samples were excluded because of the small sample size. All the following analyses were done via R version 4.0 [26] and the R-scripts are all available at the GitHub repository.

### Identification of genes involved in DNA repair pathways using REACTOME database

The 310 genes involved in 12 DNA repair pathways in Homosapiens were gathered from the Reactome database [27]. Those pathways are:

1. BER Participating Molecules [R-HSA-73884].
2. DNA Bypass Participating Molecules [R-HSA-73893].
3. DNA damage Reversal Participating Molecules [R-HSA-73942].
4. DNA double strand break response Participating Molecules [R-HSA-5693606].
5. Fanconi Anemia Participating Molecules [R-HSA-6783310].
6. HDR through HRR alone Participating Molecules [R-HSA-5693567].
7. HDR through HRR and SSA Participating Molecules [R-HSA-5693567].
8. HDR through MMEJ Participating Molecules [R-HSA-5685939].
9. HDR through SSA Participating Molecules [R-HSA-5693567].
10. MMR Participating Molecules [R-HSA-5358508].
11. NER Participating Molecules [R-HSA-5696398].
12. Non-homologous end joining Participating Molecules [R-HSA-5693571]

* Note: No proteins existed in pathway 9 only, as all the players that function in HDR through SSA, play a role in other pathways.

### Differential gene expression and miRNAs analysis

RNA-seq data obtained consisted of the expression raw counts for 60483 transcripts. The org.Hs.eg.db R package (version 3.11.4) was used to convert the Ensemble gene ID to Gene symbol resulting in 25531 genes symbols. The miRNA-seq data obtained consisted of the expression raw counts for 1881 miRNAs. In both analyses, DESeq2 R package (version 1.28.1) [28] was used to collapse the five technical replicas and run the default differential expression analysis on the datasets. DESeq2 utilizes median of ratios method to normalize gene raw counts and estimate size factors. Negative binomial generalized linear model is used for dispersion estimation to model gene read counts. Differential analysis is performed with two-tailed Wald test. The results for comparing IDC samples to normal_ductal samples, and IDC to LC samples were obtained with |log2FC| > 1 and adjusted P-value < 0.05, for both DEGs and DEMs. For the DNA repair genes, the DESeqResults object had 25177 genes. Of them, only 285 out of the 310 repair genes were found (see S3 Table for the full list of DNA repair genes, functions as per Uniprot and excluded genes). Finally, Biomart R package was used to get the description of the Genes from Ensemble database (version 2.44.1) [29]. For the miRNA differential expression analysis, DNA repair targets were validated via miRTarBase 2020 and the miRNAs binding regions on the DEGs mRNAs were obtained from TargetScan [30,31].

### Generation of protein interaction subnetworks of DNA repair related pathways

Protein-protein interactions (PPI) are retrieved from Reactome [27] and Biogrid databases [32]. In total, 18572 interactions between 4515 proteins from Reactome and 50759 interactions between 9983 proteins from Biogrid are obtained. These PPI networks are merged and resulted in a reference interactome composed of 65464 unique interactions between 11061 proteins. Proteins associated with DNA repair related pathways in Reactome, are searched for in the reference interactome. For each pathway, a subnetwork is generated. All networks are visualized in Cytoscape [33] and networks are analyzed in networkx Python package [34].

## Results

### IDC and LC have different transcriptomic profiles

**Fig 1:**
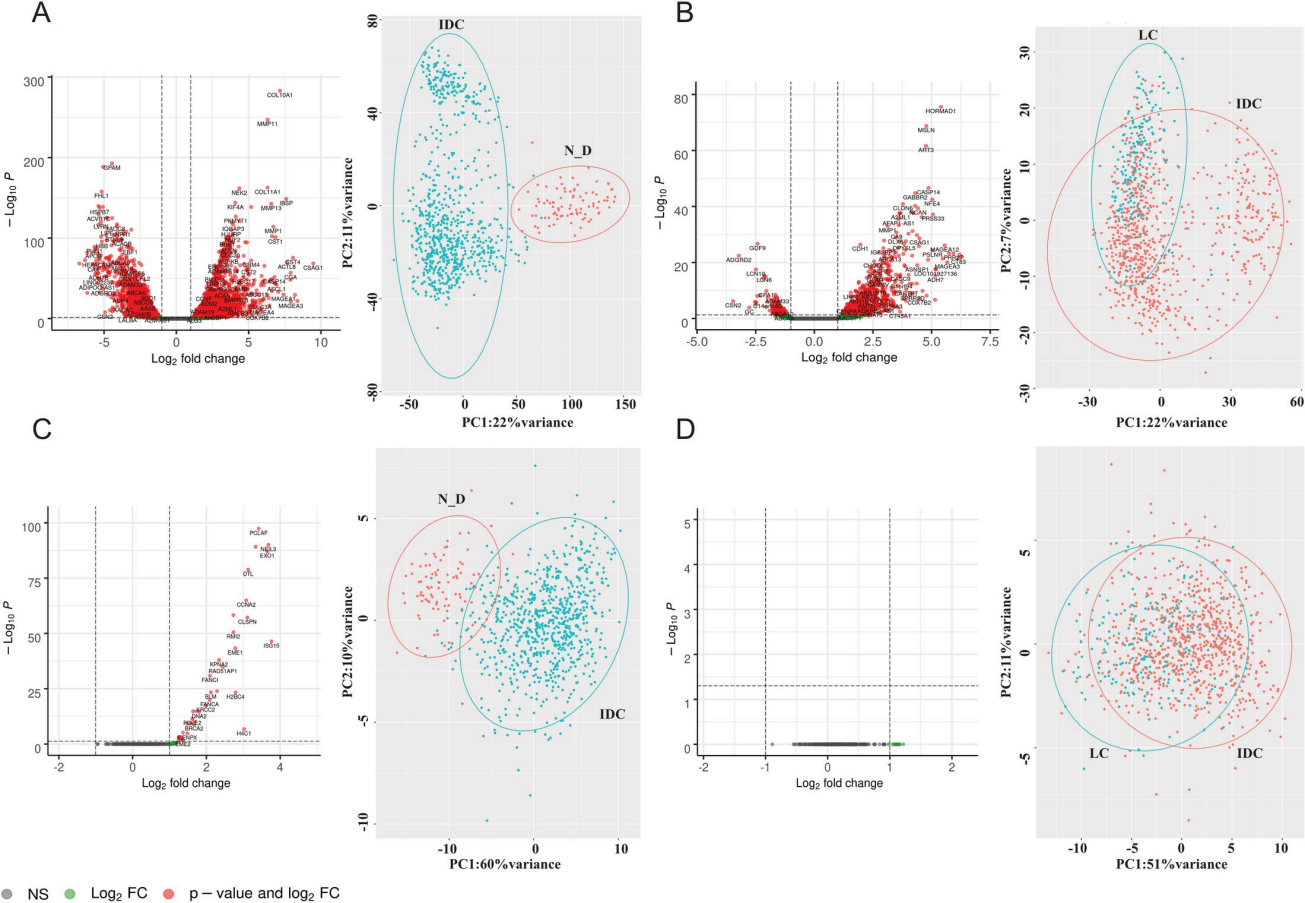
Analysis of DEGs and DNA repair DEGs in IDC and LC. A) Analysis of DEGs in IDC vs Normal_Ductal. Volcano plot of IDC vs Normal_Ductal (25,177 genes). The positive log2 fold change reflects overexpression in IDC when compared to normal_ductal (N_D), and the negative log2 fold change reflects downregulation in IDC in comparison to normal_ductal. PCA of all DEGs (3854 genes) between normal_ductal vs IDC. B) Analysis of DEGs in IDC vs LC. Volcano plot of IDC vs LC (25,177 genes). The positive log2 fold change reflects overexpression in IDC when compared to LC, and the negative values reflect the downregulation in IDC in comparison to LC. PCA of all DEGs (663 genes) between IDC and LC. C) Analysis of DNA repair DEGs in IDC vs Normal_Ductal; Volcano plot of IDC vs Normal_Ductal (285 genes). The positive log2 fold change reflects overexpression in IDC when compared to normal_ductal, and the negative log2 fold change reflects downregulation in IDC in comparison to normal_ductal. PCA of repair DEGs (36 genes) between normal_ductal vs IDC. D) Analysis of DNA repair DEGs in IDC vs LC; Volcano plot of IDC vs LC (285 genes). PCA of repair DEGs (36 genes) between IDC and LC. Color code for the volcano plots: Red; DEGs (|log2FC| > 1 and adjusted P-value < 0.05), Green; (|log2FC| > 1 and adjusted P-value > 0.05), grey; not significant (NS).

In order to determine the differentially expressed genes (DEGs) between normal ductal tissues (normal_ductal) and IDC tissues of female patients, the TCGA-BRCA dataset was utilized. The dataset includes RNA-seq data of 771 IDC and 89 normal_ductal samples. A total of 25177 genes have been analyzed and we could identify 3854 total DEGs between normal_ductal and IDC at the set cutoff of |log2FC| > 1 and adjusted P-value < 0.05 (S1 Table). The 3854 DEGs are displayed in red in the Volcano plot, where the positive log2 fold change reflects overexpression in IDC when compared to normal_ductal, and the negative log2 fold change reflects downregulation in IDC in comparison to normal_ductal. As shown, there are slightly more genes upregulated (2236) than downregulated (1618) (Fig 1A and S1 Table). The principle component analysis (PCA) representing the DEGs between the IDC and normal_ductal samples showed two separate clusters for each of IDC and LC, with almost no overlap (Fig 1A).

Previous studies analyzed the differences in the transcriptomic profiles of IDC and LC. However, the small sample size was a limitation. For example, Bertucci et al. utilized both array-CGH and cDNA microarray to analyze the DEGs between 29 IDC and 21 LC samples [35]. Zhao et al. also conducted a cDNA microarray study to analyze the DEGs between 38 IDC and 21 LC samples [5]. In our study, we analyzed the DEGs between 771 IDC and 203 LC samples from the TCGA-BRCA dataset. The utilization of RNA-Seq data, which is taking over the array approaches, in addition to analyzing a larger number of samples, should increase our ability to identify more DEGs and reach more accurate conclusions [36]. Since seven normal lobular tissues were only provided in the dataset, we directly compared both breast cancer subtypes instead of utilizing the normal tissues as a reference in each case. We identified a total of 663 DEGs between IDC and LC at the set cutoff of |log2FC| > 1 and adjusted P-value < 0.05 (S2 Table). We have a significantly larger number of upregulated DEGs (591) in IDC than downregulated (72), as shown in red in the Volcano plot; positive log2 fold change reflects overexpression in IDC when compared to LC, and the negative log2 fold change reflects the downregulation in IDC in comparison to LC (Fig 1B and S2 Table). The PCA plot shows an overlap between the samples, indicating more similarity between the two cancer subtypes in comparison to the dissimilarity between normal_ductal and IDC (Fig 1B).

### The DNA repair genes are expressed in a similar manner in IDC and LC

DNA repair genes are known to be associated with the development and progression of breast cancer. In addition, the response of breast cancer patients to therapy is usually affected by the repair machinery in the cells [37]. However, no specific analysis for DNA repair genes in IDC and LC was previously reported to our knowledge. We focused on the analysis of DNA repair genes in both subtypes, to identify novel DEGs and predict how their expression level can affect response to treatment. We obtained a list of 310 DNA repair genes from the Reactome database [27], then analyzed the differential expression of 285 genes between normal_ductal and IDC, as 25 genes were excluded (see methods). The 25 excluded genes were histone genes contributing to BER (see S3 Table for the full list of DNA repair genes, functions as per Uniprot and excluded genes). We could identify 36 DNA repair genes to be upregulated in IDC when compared to normal_ductal (S4 Table and Fig 1C). The PCA plot of normal_ductal and IDC showed a small overlap, which indicates more similarity between the samples upon focusing on the DNA repair expression profile (Fig 1C). Upon comparing the DNA repair genes between IDC and LC, no DEGs were found (Fig 1D and S5 Table). The PCA plot also shows a great overlap between IDC and LC, indicating that repair genes are expressed in a similar manner in the two subtypes (Fig 1D).

**Fig 2:**
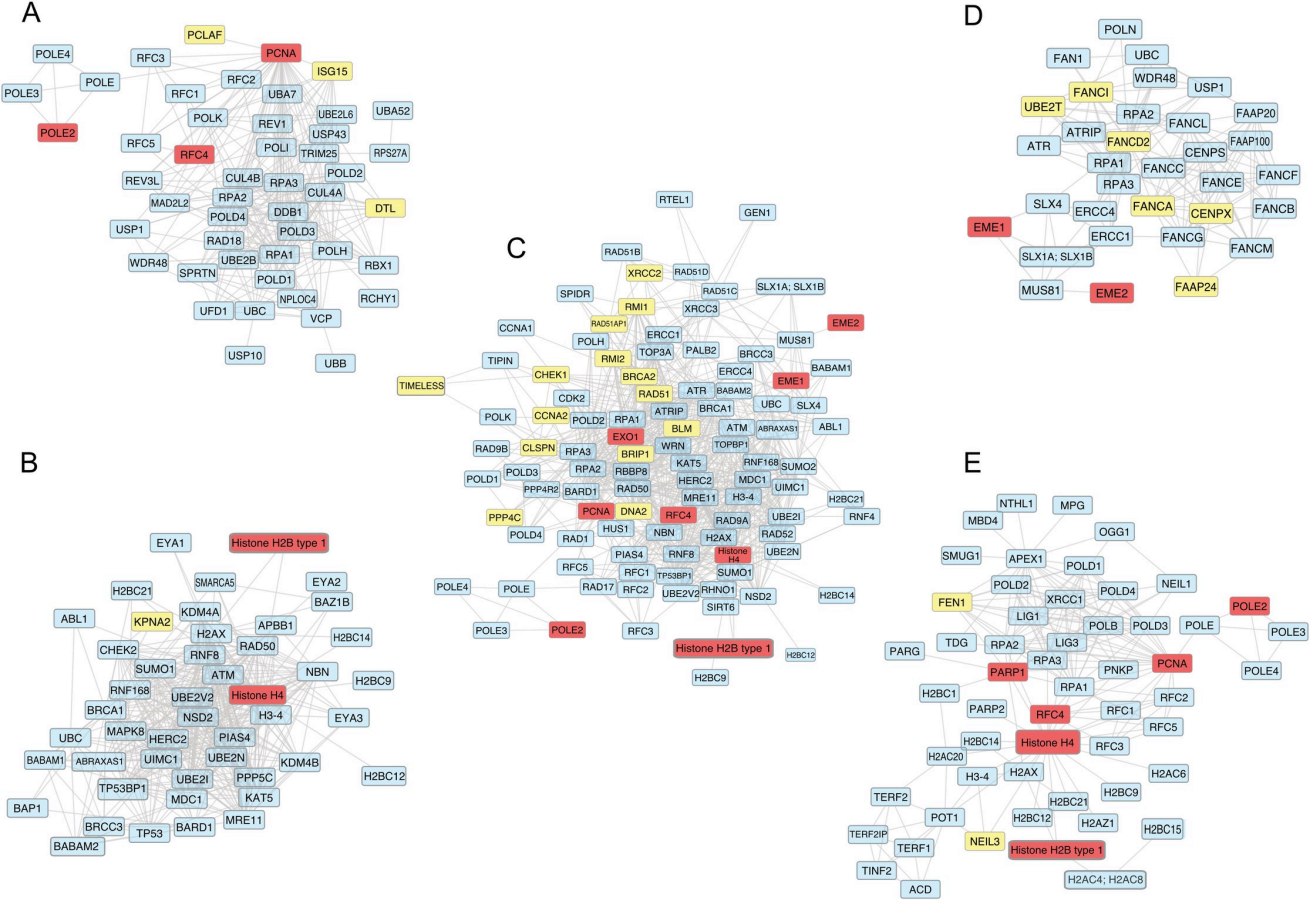
Protein-protein interaction networks of five repair pathways. A) DNA bypass B) DNA DSB response C) HDR D) FA E) BER. Overexpressed genes are highlighted in red and yellow. Red indicates that the genes function in other repair pathways besides the pathway indicated. Grey edges represent protein-protein interactions.

Our identified DNA repair DEGs function either in DNA bypass (Fig 2A), DNA double strand break (DSB) response (Fig 2B), Homology-directed repair (HDR) (Fig 2C), Fanconi anemia (FA) (Fig 2D), Base excision repair (BER) (Fig 2E) or in multiple repair pathways. The proteins that were shown to be overexpressed in both IDC and LC are highlighted in either yellow or red. Yellow indicates that the proteins function solely in the indicated DNA repair pathway and red indicates that the proteins function in multiple repair pathways (Fig 2).

**Fig 3:**
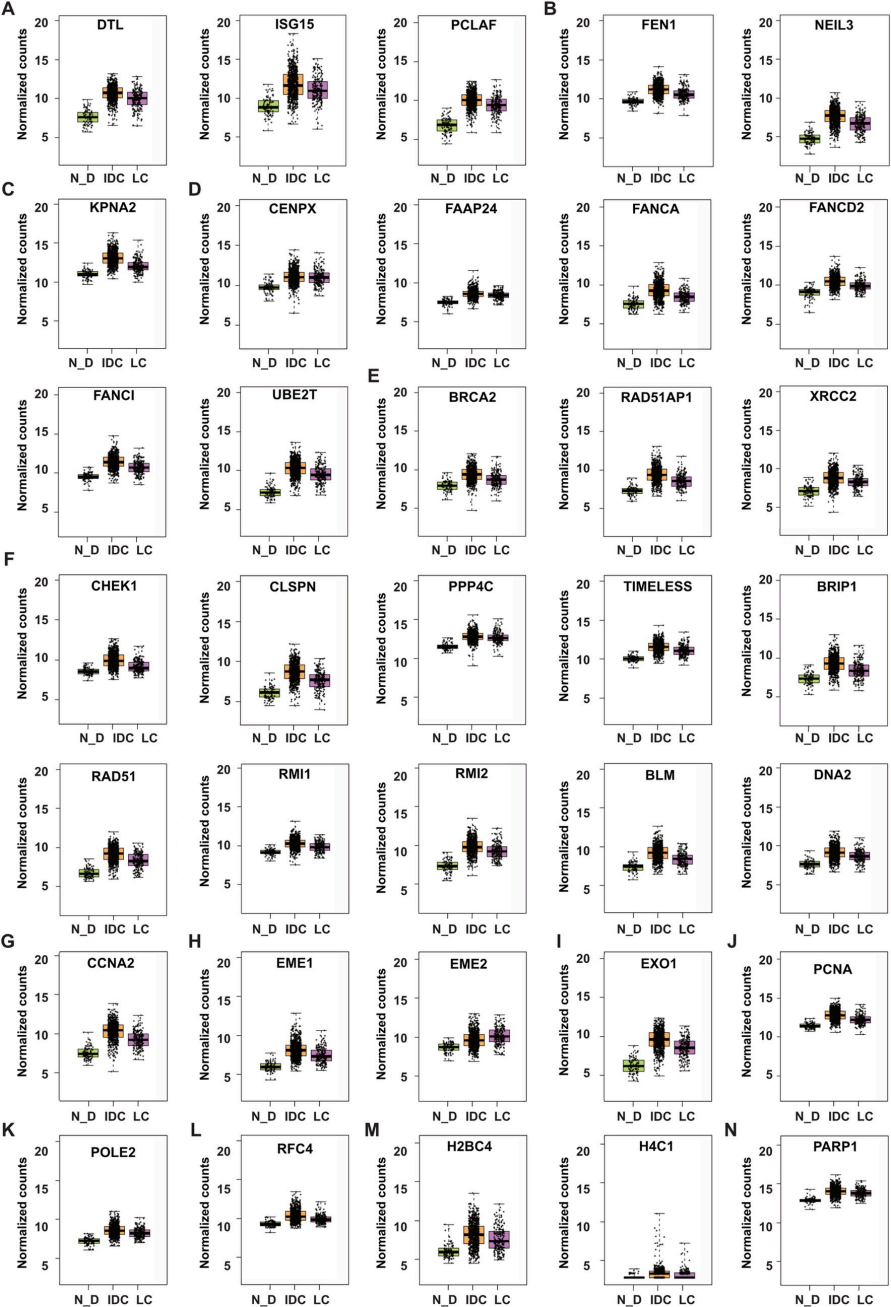
Normalized counts of the DNA repair DEGs. A) DNA bypass DEGs, B) BER DEGs, C) DNA DSB response DEG and D) FA DEGs. E-G) DEGs that function in HDR; E) through HRR&SSA F) through MMEJ and both HRR & SSA G) through MMEJ. H-N) DNA repair DEGs functioning in multiple pathways; H) FA, HDR through both HRR & SSA, I) HDR through MMEJ, HRR&SSA, MMR, J) DNA bypass, BER, NER, MMR and HDR through both HRR & SSA, K) DNA bypass, BER, NER and HDR though HRR & SSA, L) DNA bypass, BER, NER and HDR through MMEJ and both HRR&SSA, M) BER, NHEJ, DNA DSB response, HDR through both MMEJ, HRR & SSA and N) BER and NER. Boxplots represent normalized counts in normal_ductal ‘N_D’ (Green), IDC (Orange) and LC (Purple).

For better visualization of the expression levels of the DNA repair DEGs in different individuals, we combined the normalized counts for each DEG in normal_ductal, IDC and LC samples in individual box plots (Fig 3). The overexpressed genes include bypass genes; DTL, ISG15 and PCLAF (Fig 3A), BER genes; FEN1 and NEIL3 (Fig 3B), KPNA2, the DNA double strand break (DSB) response (Fig 3C), FA genes; CENPX, FAAP24, FANCA, FANCD2, FANCI and UBE2T (Fig 3D). The HDR players are represented in Fig 3E–3G. BRCA2, RAD51AP1 and XRCC2 that function in HDR through both HRR & SSA are represented in Fig 3E. CHEK1, CLSPN, PPP4C, TIMELESS, BRIP1, RAD51, RMI1, RMI2, BLM and DNA2 functioning though MMEJ/alternative non-homologous end joining (alt-NHEJ) and both HRR & SSA are represented in Fig 3F and CCNA2 functioning through MMEJ is represented in Fig 3G. Finally, the overexpressed genes functioning in multiple repair pathways are EME1, EME2 (Fig 3H), EXO1 (Fig 3I), PCNA (Fig 3J), POLE2 (Fig 3K), RFC4 (Fig 3L), H2BC4, H4C1/Histone 4 (Fig 3M) and PARP1 (Fig 3N). For all repair DEGs, a remarkable variation between the normalized counts in different individuals is observed. This indicates that despite the fact that a significant number of patients overexpress the 36 genes, many patients show downregulated expression or expression levels similar to the normal_IDC (Fig 3).

**Fig 4:**
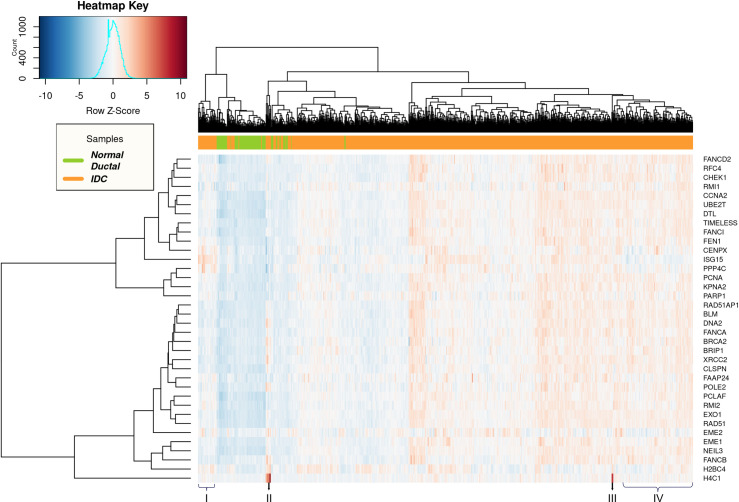
Heatmap representing the 36 DNA repair DEGs in normal_ductal vs IDC. Normal Ductal/normal_ductal (Green) and IDC (Orange).

**Fig 5:**
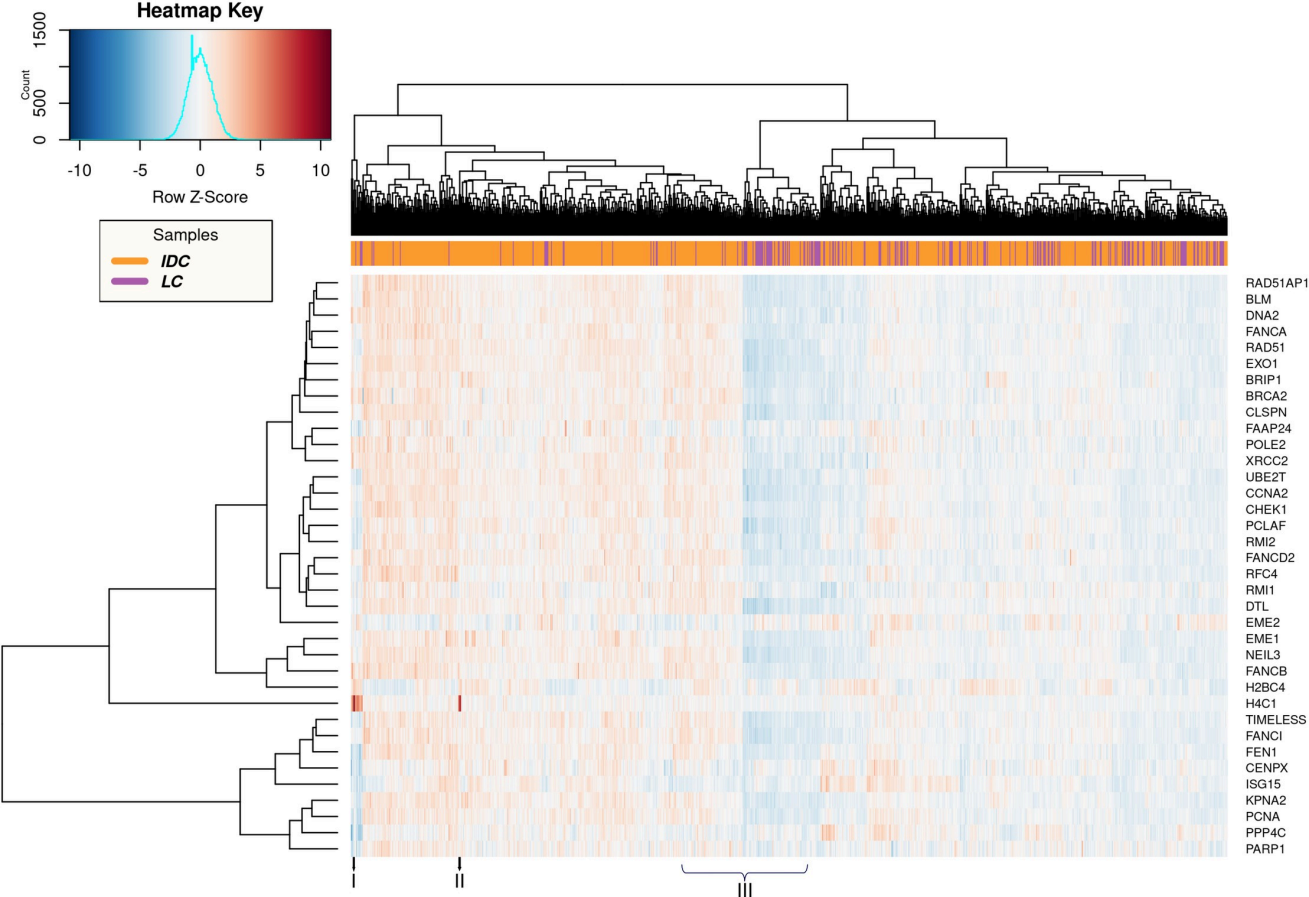
Heatmap representing the 36 DNA repair DEGs in IDC vs LC. IDC (Orange) and LC (Purple).

To focus on the individual variations, we plotted heatmaps to visualize the DNA repair DEGs across different samples analyzed (Figs 4 and 5). Upon comparing the normal_ductal and IDC samples, we observed clear subtypes clusters with a very small overlap (Fig 4). Nevertheless, a remarkable variation in the expression levels of the genes in different samples was evident. The IDC samples represented in ‘I’ were also clustered very closely to the normal samples, indicating that their expression for DNA repair genes is more similar to normal_ductal than IDC. However, CENPX, ISG15 and PPP4C were clearly overexpressed in the same samples, similar to other IDC samples and distinct from the normal_ductal. Interestingly, the IDC samples represented in ‘IV’ show downregulation of the same genes; CENPX, ISG15 and PPP4C, similar to normal_ductal. We also observed that samples represented in ‘II’ and ‘III’ show a significant overexpression for H4C1, which is distinct from normal_ductal and other IDC samples (Fig 4). Overall, the data indicates clear individual variations among patients despite having a pattern for normal_ductal and IDC, separately.

Upon comparing IDC and LC, we could not observe clear clusters for the samples, indicating a great similarity in the expression of DNA repair genes (Fig 5). Nevertheless, individual variations were also clearly observed. For example, samples represented in ‘I’ and ‘II’ show a clear overexpression of H4C1 in specific IDC patients. This indicates that this evident increase in the expression of H4C1 is specific to IDC not LC. Finally, the samples represented in ‘III’ also show a huge variation in expression levels of all repair DEGs regardless of the breast cancer subtype (Fig 5).

### Normal_ductal, IDC and LC show different miRNAs expression profiles

**Fig 6:**
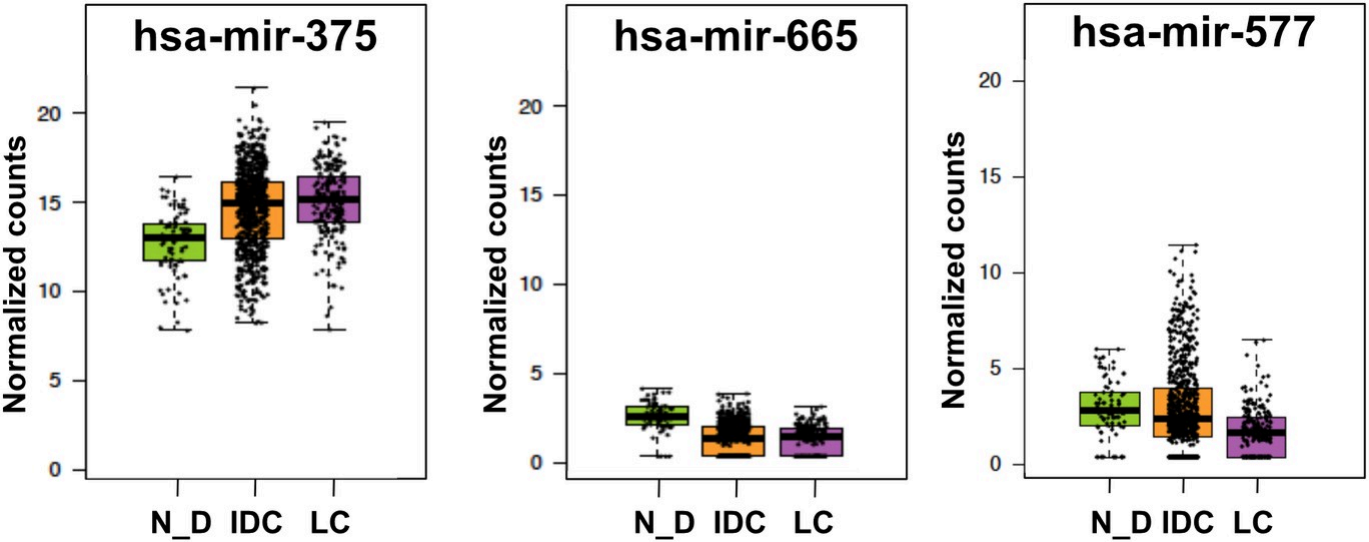
Selected DEMs that are functionally validated to target DNA repair DEGs. Boxplots represent normalized counts in normal_ductal ‘N_D’ (Green), IDC (Orange) and LC (Purple).

We have analyzed the differentially expressed miRNAs (DEMs) between 82 normal_ductal and 760 IDC samples. In addition, we analyzed the DEMs between 760 IDC and 202 LC samples. S6 and S7 Tables show 32 DEMs between normal_ductal and IDC and 7 DEMs between IDC and LC, respectively. We focused only on the miRNAs that were validated experimentally to target our DNA repair DEGs and those with predicted target sequences to infer the possible consequences of the miRNA-gene interaction (Table 1) [30,31]. We could find that hsa-miR-375 and hsa-miR-665 were upregulated and downregulated, respectively in IDC in comparison to normal_ductal (S6 Table and Table 1). No significant differences were detected for both miRNAs between IDC and LC, suggesting similar expression in both subtypes. Moreover, hsa-miR-577 was found to be upregulated in IDC in comparison to LC (S7 Table and Table 1). hsa-miR-375, hsa-miR-665 and hsa-miR-577 were validated to target EXO1, DNA2 and PARP1, respectively (Table 1) [31]. The three miRNAs target the 3’UTR of the respective mRNAs [30], which can result in either degradation, translation repression or translational upregulation in some cases [38,39]. Looking carefully at the differential expression levels of the miRNAs in different individuals, we could find a clear variation among different patients (Fig 6).

**Table 1:** Selected DEMs that target DNA repair DEGs.

miRNA	Target DNA repair Gene	miRNA binding region (TargetScan)	Expression level of miRNA	Expression level of DNA repair gene	Validation method
**hsa-mir-375**	EXO1	3’UTR	Up-regulated in tumor	Gene Up-regulated	Microarray
**hsa-mir-665**	DNA2	3’UTR	Down-regulated in tumor	Gene Up-regulated	NGS
**hsa-mir-577**	PARP1	3’UTR	Up-regulated in IDC in comparison to LC	Gene Up-regulated	NGS

The miRNA, its target DNA repair gene and the validation methods were obtained from miRTarBase 2020 [31]. The miRNAs binding regions on the DEGs mRNAs were obtained from TargetScan [30].

### Cohort analysis did not identify BRCA1 and TP53 as DEGs, despite huge individual variations

In this study, we did not use a restrictive cutoff so that we increase the number of DEGs between the samples. However, despite using a cutoff of |log2FC| > 1 and adjusted P-value < 0.05, we could not identify some DNA repair genes of known association to breast cancer; such as BRCA1 and TP53 [40–43]. Therefore, we took BRCA1 and TP53 as examples for demonstrating how cohort analysis may not identify certain genes as DEGs, while they could be potentially associated with IDC and LC (S1 Fig). Observing the distribution of the normalized counts in IDC and LC, it is evident that multiple patients have both genes downregulated and other patients show overexpression as well. The huge distribution of counts is also more evident in IDC, which contains a greater number of samples (S1 Fig). Therefore, we believe not considering BRCA1 and TP53 as DEGs in IDC and LC samples could be owed to the inter-individual heterogeneity rather than the irrelevance of the genes analyzed to IDC and LC (S1 Fig). This can also apply for many other genes that we did not focus on in this study.

## Discussion

Our transcriptomic analysis of RNA-Seq data of normal_ductal and IDC have revealed 3854 DEGs. In addition, 663 genes were differentially expressed between IDC and LC. We compared our results to the previously mentioned studies; Bertucci et al. and Zhao et al., that investigated the transcriptomic differences between IDC and LC [5,35]. Consistent with both studies, CDH1 was upregulated in IDC in comparison to LC [5,35]. Moreover, CIDEC and MFAP4 were overexpressed in LC as reported in Bertucci et al. [35], PLIN1 was overexpressed and FADS2, HIST1H2AL/H2AC11, HIST1H2BJ/H2BC11 and HIST1H3D/H3C8 were downregulated in LC as reported in Zhao et al. [5]. Nevertheless, we could find that GDPD2 is downregulated in LC, which contradicts previous reports showing its upregulation [35]. In addition, contradictory to our study, both Bertucci et al. and Zhao et al. reported that ALDH1A1, FABP4 and VWF are upregulated in LC. We found FABP4 to be downregulated in LC, while the other two genes were not differentially expressed in IDC and LC. The remaining DEGs reported by either study were also not observed in our study and vice versa (S2 Table). The discrepancies in the results can be owed to our utilization of RNA-Seq data and the larger number of samples analyzed in our study, which is most likely to our advantage as the analysis should be more reliable [36]. In addition, the utilization of different patients’ samples can also lie behind the differences observed.

Previous studies have focused on analyzing DNA repair genes in breast cancer. For example, a previous study has shown that 21 DNA repair genes were differentially expressed in Hispanic breast cancer female patients using DNA microarray [44]. In line with our results, overexpression of NEIL3, EME1, PCNA and RAD51 genes were reported, but the study did not report our other DEGs and vice versa. The study however, did not focus on IDC and LC and also focused on a specific population, which could explain the discrepancies in the results. In our study, we analyzed DNA repair genes in IDC versus (vs) LC specifically. 36 DNA repair genes were found to be overexpressed in both subtypes, which are all previously associated with breast cancer.

Here, we discuss the previously reported associations. PCLAF and ISG15 overexpression are associated with poor prognosis of breast cancer, and DTL knockdown decreases breast cancer cells’ proliferation and metastasis [45–48]. FEN1 overexpression increases breast cancer progression and its transcription is also activated following anti-cancer treatments. Therefore its inhibition is a potential approach to overcome resistance mechanisms [49–53]. NEIL3 is also overexpressed in breast invasive carcinoma and its upregulation positively correlates with the decrease in the survival of triple negative breast cancer (TNBC) patients [54–56]. KPNA2, which encodes for Karyopherin-α2 is also highly expressed in breast cancer and its expression is associated with aggressiveness, poor outcomes in addition to chemotherapy and radiotherapy resistance [57–61]. The FA genes we identified as DEGs; CENPX, FAAP24, FANCD2, FANCI, UBE2T and FANCA are all overexpressed in breast cancer [62–65]. High FANCD2 expression correlated with poor breast cancer patients’ outcomes. However, 10–20% of breast cancer patients also show loss of FANCD2 expression [66]. Finally, UBE2T knock-down also suppresses tumor growth [62].

Germ-line mutations in BRCA2 act as a major cause of hereditary breast cancer and increase the risk of its early-onset [16,67]. The BRCA2 mutation frequency was reported to be 1–3% in breast cancer [43]. RAD51AP1 is overexpressed in breast cancer stem cells population (BCSCs) and its knockout/down reduced cancer stem cells population in breast, lung, and colon cancer mouse models and improved chemotherapy and radiotherapy [68,69]. XRCC2 is a breast cancer susceptibility gene [70]. Contradictory to our data, XRCC2 was reported to be downregulated in lymph node metastatic breast cancer tissues [71].

The overexpression of CHEK1 was postulated to lead to tumor development and a risk factor in prognostics [72]. In ER- and TNBC, CHEK1 leads to shorter survival following chemotherapy, and its absence was correlated with better outcome [73]. Thus, Chk1 inhibitors such as Prexasertib might serve as a promising therapeutic agent [74,75]. Overexpression of CLSPN coding for Claspin was reported in ER- and/or PR- breast cancer [76]. Its overexpression is a radiotherapy resistance marker in metastatic lung cancer [77], which could be potentially applicable for IDC and LC as well. Timeless promotes breast cancer progression and contributes to poor prognosis [78]. ER alpha-positive (ERα+) breast cancer relapsed patients treated with tamoxifen overexpress Timeless, suggesting its contribution to tamoxifen resistance [79]. Inhibition of the overexpressed PPP4C in breast cancer increases cisplatin sensitivity [80]. Furthermore, RAD51 overexpression positively correlates with tumor grading in IDC, but an inverse relationship was found with estrogen-receptor status [81]. Mutations in BRIP1 increases the risk of breast and ovarian cancer, and its impairment accounts for some breast cancer familial cases [82,83]. Moreover, it is overexpressed in IDC followed by LC [84]. RMI2 is also upregulated in breast cancer [85] and SNPs in BLM and RMI1 were associated with breast carcinoma [86]. Controversially, BLM was reported to be overexpressed in aggressive clinicopathological breast cancer phenotypes, and also reported to be downregulated in other breast cancer subtypes. Therefore, BLM could be a promising biomarker for subtype identification [87]. DNA2 overexpression was reported in a breast cancer cohort with significant higher expression in basal-like breast cancer more than other subtypes, and it positively correlated to metastasis. Additionally, DNA2 partial depletion decreases breast cancer tumorigenicity [88]. CCNA2 was also reported to be overexpressed in breast cancer and utilized as a prognostic biomarker for ER+ subtype. CCNA2 overexpression is correlated with anti-estrogen tamoxifen drug resistance that is usually used for treating ER+ patients. However, CCNA2 repression to reverse the tamoxifen resistance or prevent it is still under study [89,90].

EME1 Ile350Thr variant in Southern Chinese females is significantly associated with susceptibility and early onset of breast cancer and its overexpression is suggested to lead to cisplatin resistance [91,92]. Additionally, EME2 is differentially expressed in tamoxifen resistant breast cancer cells and associated with poor outcomes in patients who did not receive radiotherapy, but not in patients who were subjected to radiotherapy [93]. EXO1 polymorphism is linked to breast cancer susceptibility and the expression level is associated with poor prognosis [94,95]. EXO1 inhibitors could diminish the repair of IR-induced DSBs, which possibly improve radiotherapy and chemotherapy [96]. PCNA is usually known as a proliferating marker in breast cancer. However, there is no evidence for correlation of its overexpression with breast cancer progression [97]. Nevertheless, PCNA expression is a biomarker for predicting high risk of relapse in patients with lymph node-negative breast cancer [98]. A unique acidic form of PCNA was revealed to be present in malignant breast cancer due to post translation modification alteration [97]. An AATT deletion in intron 18 of the POLE2 subunit of Polymerase ε is associated with breast cancer, and POLE2 is suggested to contribute to lapatinib resistance in HER2+ breast cancer patients with acquired lapatinib resistance [99,100]. Furthermore, RFC4 overexpression contributes to the development of breast cancer [72]. H2BC4 and H4C1 are also overexpressed in breast cancer and the first is overexpressed in its metastatic relapse [101,102]. Finally, Poly-ADP-ribose polymerase 1 (PARP1) was shown previously to be upregulated in breast cancer, acting as an independent biomarker for poor prognosis [103].

Overall, our identified DNA repair DEGs show strong association to breast cancer. But here we report their overexpression in both IDC and LC breast cancer subtypes in particular. Rad51, NEIL3 and BLM, which were linked to invasive breast cancer and BRIP1 that was associated with IDC and LC, are the only genes to our knowledge that were previously linked to invasive breast carcinoma [81,84]. To confirm that most of the transcripts of the 36 genes are functional variants that can potentially impact cellular functions upon their overexpression, we gathered the simple somatic mutations (SSMs) data for the 36 genes in the TCGA-BRCA cases of the TCGA project (S8 Table). The data clearly indicates that SSMs are rare in the samples analyzed for SSMs; 986 out of 1098. On the contrary, the CNV gains are high for our genes. The overexpression of the genes in some of the cases analyzed can be owed to the CNV gain. Interestingly, for some genes there were a significant number of patients who suffer from CNV loss. This could explain the individual variations we have seen upon differential expression analysis.

Regarding the miRNA expression profiles in normal_ductal, IDC and LC, we believe reaching a conclusion from cohort analysis is very difficult. Analysis of their expression in individual patients will provide much more accurate conclusions. Previous studies have also reported variable expression levels in breast cancer when it comes to the DEMs represented in Table 1. For miR-375, its downregulation suppresses epithelial-to-mesenchymal transition in invasive basal-like tumor cells and its upregulation augments cellular proliferation in ERα^+^ breast cancer cells through a positive feedback loop [104–107]. miR-665 was also reported to be downregulated in breast cancer, but a recent study reported its high expression in IDC validated by microarray analysis of patients’ samples and qPCR analysis of IDC cell lines (MCF-7, MDA-MB-415, and ZR-75-30). Its upregulation promotes tumor progression through increased proliferation, cell growth and inhibition of apoptosis [108,109]. A previous study identified PARP1 as a target for miR-577 causing its down regulation [110]. In this study, we find PARP1 to be upregulated in IDC in spite of the upregulation of miR-577 (Table 1). Opposite to our findings, miR-577 was previously reported to be downregulated in breast cancer. The low levels of miR-577 were associated with increased invasiveness [111]. The differential expression of miR-577 between IDC and LC could also be attributed to variability in expression levels in the patients’ samples. Interestingly, multiple IDC patients show a remarkable overexpression of the miR-577 when compared to LC patients (Fig 6).

### Implications of overexpression of DNA repair genes on precision medicine for IDC and LC patients

The implementation of precision medicine in cancer treatment has gained a great attention in recent years. Despite the advances in developing anti-cancer drugs and the clinical success and effectiveness of certain drugs, some cancer types do not respond to treatment [112]. As a matter of fact, available therapies are actually limited to some patients with certain tumors, where less than 50% of the patients show responsiveness to around 90% of the drugs. IDC and LC also differ in response to neoadjuvant, with LC being less sensitive [4,113–115]. Nevertheless, similar treatment is usually administered to stage-matched LC versus IDC. Studying the transcriptomic profile of both subtypes is thus essential to identify the genes that contribute to the development, progression and resistance of these distinct subtypes. In addition, tailor better therapy for each subtype.

DNA repair-based targeted therapy for breast cancer induces cell death through impairing DNA repair pathways and increasing the accumulation of DNA damage and breaks. Targeted therapy has less off-target side effects and greater sensitivity than chemotherapy and radiation [116]. Some of our DEGs are known to be very important drug targets for breast cancer patients. For example, PARP inhibitors (PARPi) serve as an example of the targeted therapy [117]. PARPi have shown effectiveness in tumors with deficiencies in our DEGs such as RAD51, FANCD2, FANCA, CHK1 and XRCC2 [118–120]. For BRCA1/2 mutant breast cancers, PARPi olaparib and talazoparib are now FDA-approved monotherapies [119,121,122]. Other studies also showed that their effectiveness extend to tumors without BRCA-mutations [117,123]. A potential therapeutic approach would be combining PARPi with specific inhibitors for our DEGs to achieve better response.

As previously discussed, deciphering the DNA repair expression profile for each patient is essential to understand the cellular consequences of the therapy. However, most studies follow cohort analysis using standard statistical algorithms to determine DEGs, where various normalization methods followed by negative binomial distributions or Poisson are utilized to model the gene count data. Cutoff score based on P-value generated by statistical modeling is then applied along with expression change threshold [124,125]. This method of analysis has been successful in different ways, as they could identify biomarkers and prognostic markers and determine which genes are usually overexpressed or downregulated in certain cancer types [126]. However, the drawback of this approach is that focusing on average expression levels across patient samples is of clinical relevance to many patients; but not all. Therefore, the individuals whose expression levels can be thought of as outliers in the dataset, may not be diagnosed correctly and will not respond to some of therapies that will work for others. Recently, other analysis approaches have evolved to address such limitations where the focus is on the individual level or what is known as ‘single-subject analysis (SSA)’. SSA has been performed through utilizing either a specific individual sample versus a cohort of reference samples or a paired sample; tumor and control from the same subject [127]. Rankcomp is an example of the first method, where a ranking methodology and pairwise comparison is performed to ensure stable ranking against normal samples cohort. Then a Fisher’s exact test is utilized to detect DEGs [128]. However, Rankcomp can result in high false discovery rate [129]. PenDA is another analysis technique developed to avoid high false discovery [129]. Some of the already developed techniques for cohort analysis have also been evaluated for paired sample analysis such as EdgeR and DESeq [130]. A more advanced approach that aims at better specificity is single cell analysis via single-cell RNA sequencing (scRNA-seq). Unlike bulk analysis, which reports the predominant malignant clone, scRNA-seq has enabled the identification of the cellular heterogeneity and transcriptomic changes present in tumor microenvironment, either between the patients of same cancer types or even within the same patient [131,132]. These individualized approaches are very effective in determining the appropriate therapy for patients, and ensure the patient’s response to treatment and reduce resistance.

## Conclusion

Overall, our data indicate that LC and IDC have different transcriptomic profiles, while they express DNA repair genes in a more similar manner. The cohort analysis performed in this study identifies 36 DNA repair genes to be overexpressed in a significant number of IDC and LC patients, which could significantly affect patients’ response to specific therapy. However, the findings can be used as an indicator for the changes in transcription of a specific gene in a certain disease subtype, but not for reaching general conclusions on patients of a specific cancer type. The exact transcriptomic profile of each patient should be taken into consideration to decide for the appropriate therapy and foresee whether the patient will respond to the therapy. We suggest that a great attention should be given to the transcriptomic changes in DNA repair genes, and not only the changes on the genomic level. This should be done through utilizing the appropriate Next generation sequencing diagnostic panels, to reach the most accurate conclusions for individual patients.

## Supporting information

S1 Fig The expression of BRCA1 and TP53 in normal_D, IDC and LC. Boxplots represent normalized counts in normal_ductal ‘N_D’ (Green), IDC (Orange) and LC (Purple).
(TIFF)

S1 Table DEGs in IDC vs Normal_Ductal. The positive log2 fold change reflects overexpression in IDC when compared to Normal_ductal, and the negative values reflect the downregulation in IDC in comparison to Normal_ductal.
(XLSX)

S2 Table DEGs in IDC vs LC. The positive log2 fold change reflects overexpression in IDC when compared to LC, and the negative values reflect the downregulation in IDC in comparison to LC.
(XLSX)

S3 Table The list of DNA repair genes analyzed. The function of each gene was determined according to Uniprot. and the DNA repair pathway the protein is involved in was determined via Reactome.
(XLSX)

S4 Table DNA repair DEGs in IDC vs Normal_Ductal. The positive log2 fold change reflects overexpression in IDC when compared to Normal_ductal, and the negative values reflect the downregulation in IDC in comparison to Normal_ductal.
(XLSX)

S5 Table DNA repair DEGs in IDC vs LC. The positive log2 fold change reflects overexpression in IDC when compared to LC, and the negative values reflect the downregulation in IDC in comparison to LC.
(XLS)

S6 Table DEMs in IDC vs Normal_Ductal. The positive log2 fold change reflects overexpression in IDC when compared to Normal_ductal, and the negative values reflect the downregulation in IDC in comparison to Normal_ductal.
(XLSX)

S7 Table DEMs in IDC vs LC. The positive log2 fold change reflects overexpression in IDC when compared to LC, and the negative values reflect the downregulation in IDC in comparison to LC.
(XLSX)

S8 Table Simple somatic mutations and copy number variations in the TCGA-BRCA project cases. (XLSX)
