# Supplementary figures and images for: The overexpression of DNA repair genes in invasive ductal and lobular breast carcinomas: Insights on individual variations and precision medicine
Source: PLoS One. 2021 Mar 4;16(3):e0247837. doi: 10.1371/journal.pone.0247837 (PMC7932549; doi:10.1371/journal.pone.0247837)

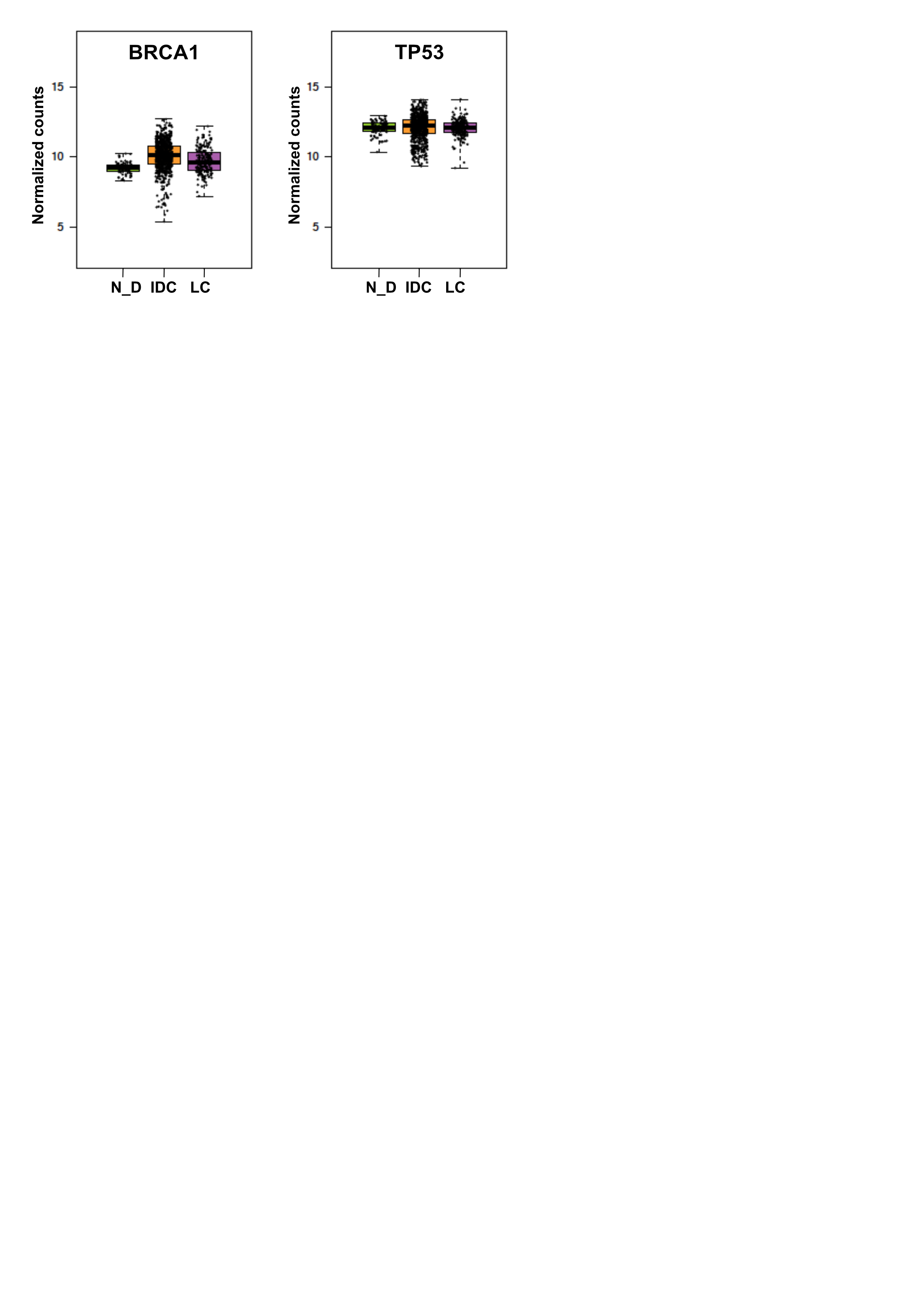

Supplement: S1 Fig — Boxplots represent normalized counts in normal_ductal ‘N_D’ (Green), IDC (Orange) and LC (Purple). (TIFF) [file pone.0247837.s001.tiff]
